# Torque expression in self-ligating orthodontic brackets and conventionally ligated brackets: A systematic review

**DOI:** 10.4317/jced.53187

**Published:** 2017-01-01

**Authors:** Yousef Al-Thomali, Roshan-Noor Mohamed, Sakeenabi Basha

**Affiliations:** 1 MDS, Dean, Faculty of Dentistry, Department of Orthodontics, Taif University, Taif, KSA; 2 MDS, Assistant Professor, Department of Pediatric Dentistry, Faculty of Dentistry, Taif University, Taif, KSA; 3 MDS, PhD, Assistant Professor, Department of Community Dentistry, Faculty of Dentistry, Taif University, Taif, KSA

## Abstract

**Background:**

To evaluate the torque expression of self ligating (SL) orthodontic brackets and conventionally ligated brackets and the torque expression in active and passive SL brackets.

**Material and Methods:**

Our systematic search included MEDLINE, EMBASE, CINAHL, PsychINFO, Scopus, and key journals and review articles; the date of the last search was April 4th 2016. We graded the methodological quality of the studies by means of the Quality Assessment Tool for Quantitative Studies, developed for the Effective Public Health Practice Project (EPHPP).

**Results:**

In total, 87 studies were identified for screening, and 9 studies were eligible. The quality assessment rated one of the study as being of strong quality, 7 (77.78%) of these studies as being of moderate quality. Three out of 7 studies which compared SL and conventionally ligated brackets showed, conventionally ligated brackets with highest torque expression compared to SL brackets. Badawi showed active SL brackets with highest torque expression compared to passive SL brackets. Major and Brauchli showed no significant differences in torque expression of active and passive SL brackets.

**Conclusions:**

Conventionally ligated brackets presented with highest torque expression compared to SL brackets. Minor difference was recorded in a torque expression of active and passive SL brackets.

** Key words:**Systematic review, self ligation, torque expression, conventional ligation.

## Introduction

Torque is defined as the twisting of a structure about its longitudinal axis, resulting in an angle of twist. It is a shear-based movement that causes rotation (1). In orthodontics, it represents the labiolingual crown/root inclination of a tooth, and it is an orthodontic adaptation used to describe rotation perpendicular to the long axis of the tooth (2,3). In clinical orthodontics, optimal labiolingual inclination of both posterior and anterior teeth is considered essential to establish a proper occlusal relationship, an esthetic smile line, proper movements of root, and subsequently long-lasting stability of the orthodontic outcome (1-4). The extent of change in the labiolingual inclination of the crowns that is torque expression depends on the stiffness or the resilience of the wire cross section, wire size, edge bevel and manufacturer tolerance, bracket slot size, engagement angle of the wire in the bracket slot, bracket placement as related to tooth morphology, mode of ligation, experimental measurement technique, and inclination of the tooth (3,4). The wide array of combinations of altering factors in defining torquing moments make the empirical clinical determination of the appropriate torquing method a difficult task for the practicing professional.

The term self-ligation in orthodontics implies that the orthodontic bracket has the ability to engage itself to the archwire (5) and these bracket systems have a mechanical device built into the bracket to close off the edgewise slot. Two types of self ligating (SL) brackets have been developed. The active one with the ligation clip exerts a pressure on the arch wire which in turn enhances rotational control and passive ones with closing mechanism that transform the open slot to a tube. Self ligating bracket system are not new to orthodontics; in the mid-1930s, the first SL bracket, the Russell attachment, was introduced in an attempt to enhance clinical efficiency by reducing ligation time (6). Recently, many studies have been conducted using SL brackets and the reported advantages of SL brackets include increased patient comfort, improved oral hygiene, increased patient co-operation, less chair time, shorter treatment time, greater patient acceptance, reduced friction, full and secure wire ligation, anchorage conservation, improved ergonomics and longer appointment intervals (7-10).

Many individual studies have evaluated the torque expression in SL brackets with conventional ones (11-22) and comprehensive synthesis of this evidence would help the clinicians use these brackets effectively. This systematic review is intended to evaluate the quantitative effects of self ligating orthodontic brackets and conventional brackets on torque expression. It is our intention to help the clinician to better understand the variables involved in generating torque moments when selecting the appropriate brackets for torque expression.

## Material and Methods

This review was planned, conducted, and reported in adherence to PRISMA standards of quality for reporting systematic reviews and meta-analyses (23). IRB approval was not required.

-Questions

We sought to examine the torque expression of SL orthodontic brackets and conventional ligated brackets and active and passive SL brackets.

-Study Eligibility

We included studies published in English language only that investigated the torque expression of SL orthodontic brackets and conventional ligated brackets, and the studies which compared torque expression in active and passive SL brackets. Papers were excluded at this stage if they were descriptive, editorial, letter, in vivo, not investigating SL brackets, or were studying other properties of SL brackets rather than torque expression.

-Study Identification

We searched MEDLINE, EMBASE, CINAHL, PsychInfo,Educational Resources Information Center (ERIC), ISI Web of Science, and Scopus using search terms designated by an experienced research librarian, focused on the search strategy (orthodontic brackets, orthodontic wire, experimental orthodontic brackets, self ligation, active self ligation, passive self ligation, torque, torque expression). To supplement the searches, the tables of content of 4 key orthodontic journals (American Journal of Orthodontics and Dentofacial Orthopedics, Angle Orthodontics, European Journal of Orthodontics, and Journal of Orthodontics) were searched for relevant articles. No beginning date was used, and the last date of the search was April 4th, 2016. We searched for additional studies in the reference lists of all articles included.

-Study Selection

We screened all titles and abstracts independently and in duplicate for inclusion. In the event of disagreement or insufficient information in the abstract, we independently and in duplicate reviewed the full text of potential articles. The inter-rater agreement for study inclusion, as assessed using an intra-class correlation coefficient, was 0.65. Conflicts were resolved by consensus discussion between the two reviewers.

-Data Extraction

We extracted data independently and in duplicate for all variables and resolved conflicts by consensus. We graded the methodological quality of these studies by means of the Quality Assessment Tool for Quantitative Studies, developed for the Effective Public Health Practice Project (EPHPP), Canada, as adapted by Thomas *et al.* (24); however, several points did not apply to this systematic review in that it was a review of *in vitro* studies rather than randomized control trials. This tool consists of six criteria: study design (objective clearly mentioned, sample size, baseline characteristics, co-interventions), measurement method, blinding, reliability, statistical analysis (statistical analysis, confounders, level of significance), clinical significance. Each criterion was rated as strong, moderate, or weak according to the dictionary of the tool; the overall assessment of the study is determined by assessing these ratings. According to the guidelines for the tool, studies with no weak rating and four strong ratings are classified as “strong”; studies with fewer than four strong ratings and one weak rating are classified as “moderate”; and studies with two or more weak ratings are classified as “weak”. Two reviewers independently performed the assessment of the quality of the included studies. Any discrepancies in quality ratings were resolved by discussion and consensus. Validity was assessed by critically examining the torque-measuring devices and methods employed in each study.

-Data Synthesis

Two reviewers did data extraction independently for the included studies, and any discrepancies were resolved by discussion and consensus. The following data were extracted from each included study: first author, publication year, measurement device, variables measured, error measurement, tested brackets with ligation used, slot size, engagement angle used, and torque play.

## Results

-Trail flow

**Figure 1:**
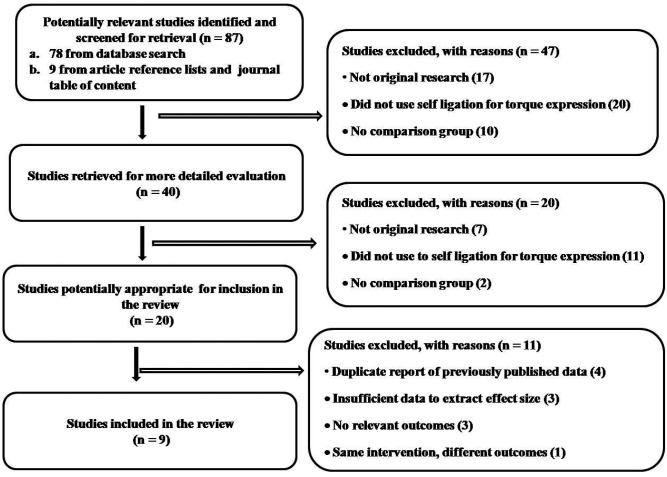
Study selection flow diagram of the systematic review.

 Using our search strategy, we identified 78 articles with an additional 9 identified from our review of references and journal indices. From these we identified 9 articles for inclusion in the present systematic review (Fig. 1).

-Study characteristics and study quality

The studies were fairly recent, with oldest study published in 2008. All of the included studies were published in English. Three (33.33%) out of 9 studies used orthodontic measurement and simulation system (OMSS) to measure torque expression. In all of the included studies, maximum mean torquing moment was measured. Two studies measured torque loss along with torque expression. The quality assessment rated one of the study as being of strong quality, 7 (77.78%) of these studies as being of moderate quality and 1 was assessed as being of weak quality ( Table 1).

**Table 1:**
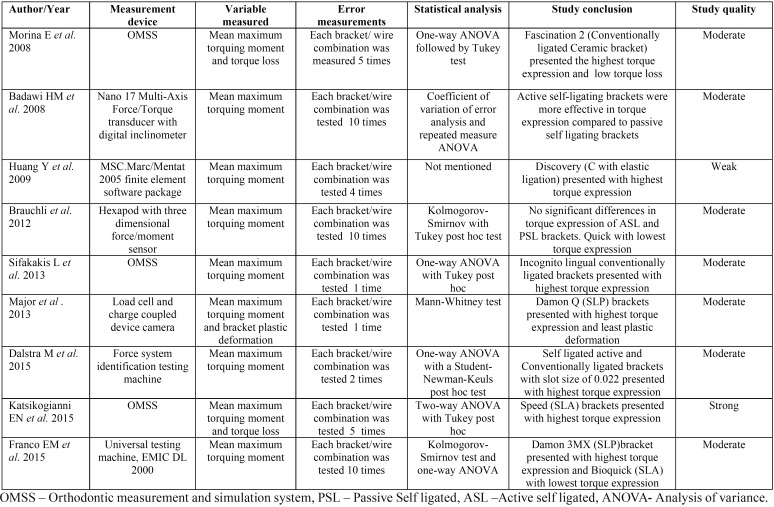
Summary of methodology, study outcome, and quality assessment of included studies.

-Tested brackets, Torque engagement angle and torque play

Seven out 9 included articles compared torque expression in self ligating and conventionally ligated brackets. Two studies compared torque expression in active and passive self ligated brackets. The slot size of use brackets varied from 0.018 to 0.022. Torque was applied on maxillary central incisor. Torque applied varies from 0.018 x 0.025 inch and 0.019 x 0.025 inch with stainless steel wire. Engagement angle varied from 4.7o to 48o. Three out of 7 studies which compared SL and conventionally ligated brackets showed, conventionally ligated brackets with highest torque expression compared to SL brackets. The results indicate that Morina *et al.* noted mean moments of 35.6 Nmm for the Fascination 2 (Ceramic, conventionally ligated with stainless steel ligation), 8.0 Nmm for the Hanson speed (ASL) bracket, and 7.8 Nmm for Damon 2 (PSL) bracket with a 0.019 x 0.025 inch stainless steel wire in a 0.022 inch slot with the OMSS. Franco *et al.* and Brauchli *et al.* recorded lowest mean torque with Quick (ASL) brackets. When active and passive self ligated brackets were compared, Badawi *et al.* showed, for the Speed (ASL) brac-kets, mean torque varied from 1.97 to 11 Nmm, for the In-Ovation (ASL) brackets, it was 3.7 – 16.7 Nmm, and for the Damon 2 (PSL) brackets, it was 2.8 – 14.2 Nmm. Major *et al.* showed, Damon Q (PSL) brackets with mean torque varied from 8.26 – 70.23 Nmm and for the Speed (ASL) brackets, it was 3.89 – 62.40 Nmm ( Table 2).

**Table 2:**
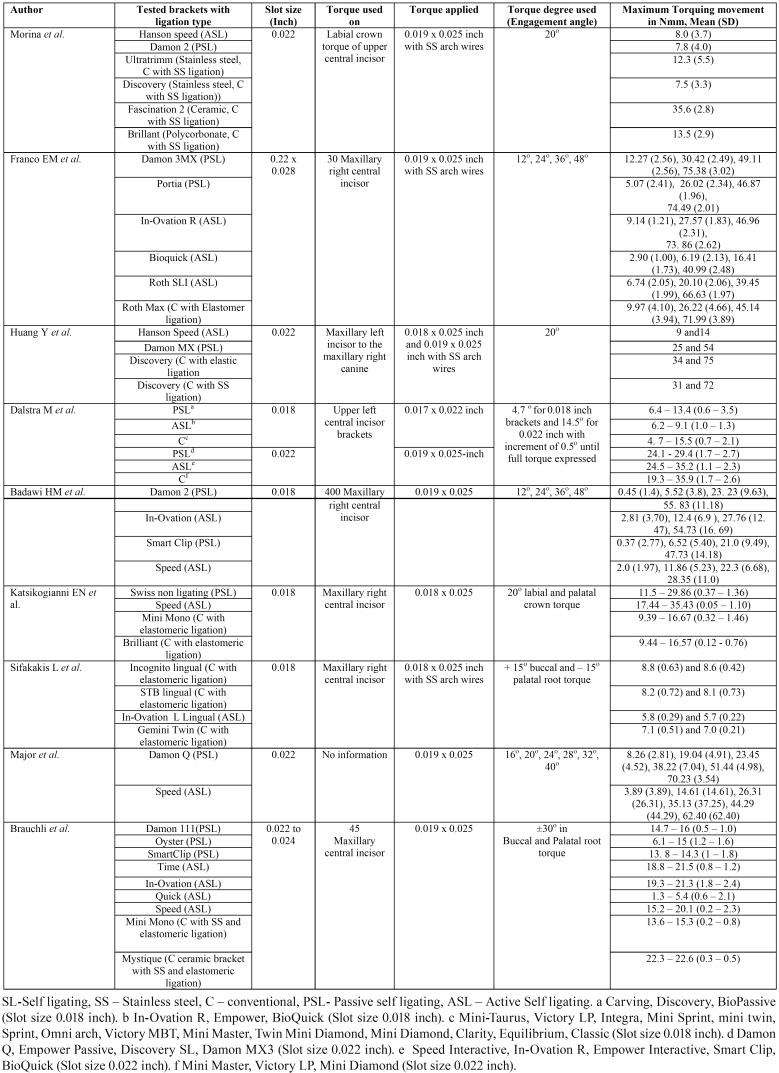
Tested brackets and Torque play of selected articles.

## Discussion

The present systematic review identified 7 *in vitro* studies in which torque expression in self ligating and conventionally ligated orthodontic brackets were compared and 2 *in vitro* studies in which torque expression in active and passive self ligating brackets were compared.

-Measurement device used to quantify torque expression

Three out of 6 studies used orthodontic measurement and simulation system (OMSS) to measure torque expression. This system comprises three forces and three moments. The sensors of the OMSS register these six components independently. The reactive moments at the centre of resistance, resulting from the leverage effect of the force application on the bracket, are also calculated by the control programme of the OMSS and entered into the simulated tooth movement. Final torque expression varied with the type of measurement device. Torque values were smaller for the OMSS experiments conducted by Morina *et al.* (3), Katsikogian-ni *et al.* (17), and Sifakakis *et al.* (18) compared to other studies which used activating experiments (11-13,16,21,22).

-Engagement angle

This parameter was tested in all of the selected studies. The engagement angle varied from 4.7o to 48o. Morina *et al.* (3) and Huang *et al.* (13) used engagement angle of 20o. However, Franco *et al.* (11) used 12o, 24o, 36o, and 48o. Dalstra *et al.* (16) used engagement angle of 4.7 o for 0.018 inch brackets and 14.5o for 0.022 inch with increment of 0.5o until full torque expressed. Results showed torque expression increased with increase in engagement angle.

-Torque expression comparison between SL and conventionally ligated brackets

In the present review, three studies showed conventionally ligated brackets presented with highest torque expression compared to self ligated brackets. Morina *et al.* (3) showed Fascination 2 (Conventionally ligated Ceramic bracket) with maximum torquing movement of 35.6 Nmm and Huang *et al.* (13) showed Discovery (conventional bracket with elastic ligation) with maximum torquing movement of 75 Nmm during insertion of 0.019 x 0.025 inch stainless steel archwire into a 0.022-inch slot at 20o engagement angle. The high torque expression of conventionally ligated brackets may be attributed to the highest raw material modulus of elasticity and increased roughness of the slot walls arising from the manufacturing process. Morina *et al.* (3) showed SL brackets with 100% torque loss compared to conventional ceramic brackets. Extended torque loss in SL brackets may complicate the treatment outcome by altering the axial inclination of maxillary anterior teeth. The study by Franco *et al.* (11) showed Damon 3MX, self ligating passive bracket presented with highest torque expression and Bioquick, self ligating active with lowest torque expression. It is generally accepted in literature that a minimum torque of 5-20 Nmm (3,11-22) is required for clinical significance. With an engagement angle of 20o and above, all type of brackets presented with the minimum clinically relevant torque. Sifakakis *et al.* (18) studied the torque expression of conventionally ligated lingual brackets and SL brackets. The lowest torque expression was observed at the SL lingual brackets (5.8 Nmm) compared to conventionally ligated lingual brackets (8.8 Nmm).

-Active versus passive self ligating brackets in torque expression

Two out of 9 studies showed active self ligating (ASL) brackets presented with higher torque expression than the passive self ligating (PSL) brackets. This happens due to the fact that the clip constantly presses the wire against the bracket slot, especially as the diameter of the arch increases, thereby resulting in better accuracy of orthodontic movement (12). According to Badawi *et al.* (12) the torque started to be expressed at 7.5o of torsion for ASL brackets and at 15o of torsion for the PSL brackets. Clinically effective torque of 5-20 Nmm was expressed at 15o to 31o of torsion for ASL brackets, and at 22.5o to 34.5o of torsion for PSL brackets. Morina *et al.* (3), Brauchli *et al.* (22) and Major *et al.* (21) found only minor differences with regards to torque expression of active and passive ligating brackets. It is important to remember that there are many factors that influence torque expression during orthodontic treatment: thickness of wire, torsion magnitude, positioning of bracket and tooth, slot size, wire and bracket composition, width and depth of the slot, brackets and wires manufacturing tolerance, difference of constituent leagues of the wires, manufacturing process of brackets (injection-molding, casting, or milling). All these elements can change the torque expressed in the bracket (11-22). Thus, it cannot be said that the active SL brackets, by itself, can effectively increase torque. Franco *et al.* (11) and Brauchli *et al.* (22) showed Quick (ASL) bracket with lowest torque expression. Clinically effective torque of 5-20 Nmm was expressed at an angulation of > 30o. This was probably due to a construction with very low torquing rails.

## Conclusions

Torque expression is a key element to obtain good results in clinical orthodontics. Accurate torque is essential to establish proper occlusion and esthetic for orthodontic treatment. The present systematic review concludes that conventionally ligated brackets presented with highest torque expression compared to SL brackets. Minor difference was recorded in torque expression of active and passive SL brackets. The torque expression increased with increase in engagement angle and a minimum torque of 5 - 20 Nmm is achieved with engagement angle of 14o or more. The torque expression increased with increase in slot size. This information helps the clinician to select the brackets of appropriate ligation and slot size with proper engagement angle to obtain good results in clinical orthodontics.
